# Isovaleric Acidemia: A Case Report

**DOI:** 10.7759/cureus.49362

**Published:** 2023-11-24

**Authors:** Elizabeth Zegarra Buitron, Daniel A Vidal Panduro, Nancy S Guillén Ramírez, María González Arteaga

**Affiliations:** 1 Internal Medicine, School of Medicine, Universidad Peruana de Ciencias Aplicadas, Lima, PER; 2 Paediatrics and Child Health, Hospital Nacional Docente Madre Niño San Bartolomé, Lima, PER; 3 Paediatrics and Child Health, Hospital Nacional Docente Madre Niño San Bartolomé, LIMA, PER

**Keywords:** chronic intermittent isovaleric acidemia, leucine metabolism, metabolic acidosis, organic acidemia, isovaleric acidemia

## Abstract

Isovaleric acidemia is an autosomal recessive disease of leucine metabolism. The clinical presentation is variable and three phenotypes are described, asymptomatic, acute neonatal, and chronic intermittent. Infections are the most important trigger for catabolic crises. Diagnosis is based on the detection of isovalerylglycine CoA in urine and elevated levels of isovaleryl (C5) carnitine in blood. Long-term treatment consists of prevention of catabolic state, dietary restriction, and supplementation with L-carnitine and/or L-glycine. We present the case of a three-year-old female patient with multiple episodes of decompensation since the age of two years. The episode in which she was diagnosed had encephalopathy, with no neurological sequelae. Currently, the patient continues with dietary restrictions and supplementation with good nutritional and growth results for her age.

## Introduction

Isovaleric acidemia (IVA) is an autosomal recessive disorder of leucine metabolism [1] caused by a deficiency of isovaleryl-CoA dehydrogenase (IVD), an enzyme involved in the leucine catabolic pathway, which catalyzes the conversion of isovaleryl-CoA to 3-methylcrotonyl-CoA [1-4]. Deficiency of this enzyme results in the accumulation of high concentrations of isovaleric acid and its derivatives, such as isovalerylglycine (IVG), isovaleryl (C5) - carnitine, and 3-hydroxyisovaleric acid in blood and urine, which could result in a potentially fatal metabolic encephalopathy [2-6]. The incidence of this disease in the United States is 1 in 250,000 and in China, it is 1 in 190,000 [2].

The clinical presentation of this disease varies from an asymptomatic presentation to severe involvement with significant neurological sequelae and even death [2,4]. Three phenotypes of this disease have been described: acute neonatal, which presents shortly after birth; chronic intermittent, associated with developmental delay; and asymptomatic, which is detected by newborn screening (NBS) with the presence of a lower accumulation of isovaleric acid and its derivatives [6]. Acute metabolic decompensation can occur during a catabolic state, with infections being the most frequent trigger [1,3,7]. It is important to emphasize that early diagnosis, adequate treatment, and prevention of new episodes of decompensation are crucial to reduce mortality and avoid neurocognitive sequelae [2,6].

We present the case of a three-year-old female patient with multiple episodes of decompensation, one of them with encephalopathy and seizures.

## Case presentation

A female patient born at term, weighing 2900 grams, APGAR (appearance, pulse, grimace, activity, and respiration) 9-9-10, with no prenatal history, presented neonatal pneumonia at two days of life, for which she was hospitalized for seven days, with complete recovery. The parents were apparently healthy, had no relevant medical history, and denied any blood relationship between them. She did not present intercurrences until she was two years old.

Since two years of age, the patient presented episodes of repetitive vomiting with moderate to severe dehydration, was treated in other hospitals in the country, and presented a complete recovery. Between each episode, the patient was asymptomatic and in good health.

At three years of age, the patient was admitted to the emergency room of the San Bartolomé Hospital in Lima, Peru, with a 10-hour illness, for presenting repeated vomiting, approximately 10 (1/4 cup), of food content, associated with drowsiness, with no other symptomatology. It should be noted that prior to her admission, the patient had a nutritional diagnosis of exacerbated chronic malnutrition. However, her psychomotor and neurological development were appropriate for her age.

An arterial blood gas (ABG) analysis was performed, showing metabolic acidosis. A urine test was also performed showing ketonuria 1+. Metabolic acidosis was corrected, and the patient was treated with intravenous fluids and antiemetics. During her hospitalization, bilious vomiting persisted, but less frequently. An upper gastrointestinal endoscopy was performed in another health facility, with a diagnosis of esophagitis and erosive gastritis. Likewise, brain magnetic resonance imaging with contrast with 3D spectroscopy was performed, in which no pathological findings were evidenced.

Five days after admission, the patient was found to have bradycardia and was also found to be excessively drowsy; the last episode of vomiting was recorded two days before this event. Laboratory tests were performed and showed respiratory alkalosis and low sodium and potassium levels (Table 1). Due to electrolyte disturbances, bradycardia, and excessive drowsiness, the patient was admitted to the pediatric intensive care unit (PICU).

**Table 1:** Comparison of laboratory results, consciousness, and heart rate between the first and fifth days of patient admission ABG - arterial blood gas; pH - potential of hydrogen (measures the acid-base balance of the blood); PaCO2 - arterial partial pressure of carbon dioxide; PaO2 - arterial partial pressure of oxygen; HCO3 - concentration of bicarbonate in arterial blood; K+ - potassium, Na+ - sodium; Cl- - chloride; Ca+ - calcium, bpm – beats per minute

Laboratory exams	Parameters	First day of admission	Five days after admission	Normal range
ABG analysis	pH	7.2	7.5	7.35 - 7.45
	PaCO2	24.7 mmHg	25.1 mmHg	35 - 45 mmHg
	PaO2	50.3 mmHg	12.3 mmHg	60 - 100 mmHg
	HCO3	10.9 mEq/L	22.7 mEq/L	22 - 26 mEq/L
	Anion gap	36.2 mEq/L	15.6 mEq/L	8 - 16 mEq/L
Glucose	-	50 mg/dL	103 mg/dL	70 - 100 mg/dL
Electrolytes	K+	4.2 mEq/L	1.3 mEq/L	3.5 - 5.0 mEq/L
	Na+	145 mEq/L	128 mEq/L	135 - 145 mEq/L
	Cl-	102 mEq/L	91 mEq/L	90 - 110 mEq/L
	Ca+	1.3 mmol/L	1.2 mmol/L	1.20 - 1.32 mmol/L
State of consciousness	-	Drowsiness	Drowsiness	Awake, reactive, alert
Heart rate	-	125 bpm	62 bpm	80 - 120 bpm

The patient was on mechanical ventilation for four days. During her stay in the PICU, she was diagnosed with a urinary tract infection, with a positive urine culture for Escherichia coli and Enterobacter cloacae. In addition, a chest X-ray was performed and alveolar infiltrate was observed in the right hemithorax and left apex. In addition, clonic movements of the upper limbs were reported, so an electroencephalogram was performed, with normal results and no evidence of epileptogenic foci. The patient was evaluated by a pediatric endocrinologist and geneticist who recommended testing for pyruvate, ammonium, lead, lactic acid, and beta-hydroxybutyrate, all of which turned out to be within normal limits (Table 2).

**Table 2:** Pediatric endocrinology and genetics laboratory tests

Laboratory exam	Result	Normal range
Pyruvate dosage	0.05 nmol/L	0.03 - 0.08 nmol/L
Plasma ammonium	48.50 umol/L	11.2 - 55. 3 umol/L
Plasma lead	< 1.8 ug/dL	< 3.5 ug/dL
Lactic acid	0.94 mmol/L	0.7 - 2.5 mmol/L
Beta-hydroxybutyrate	0.13 mmol/L	0.02 - 0.27 mmol/L

Additionally, a karyotype was performed, with normal results. Twenty-three days after admission, the patient was discharged in stable condition. Subsequently, the result of tandem mass spectrometry of acylcarnitines in dried blood spots showed results compatible with isovaleryl-CoA dehydrogenase deficiency (Table 3). Likewise, the result of the organic acid profile in urine, through gas chromatography-mass spectrometry, showed an increase of isovaleric glycine, compatible with the diagnosis of isovaleric acidemia. DNA analysis was performed, but no A282V mutation was detected.

**Table 3:** Tandem mass spectrometry of acylcarnitines in dried blood spots

Tandem mass	Result	Normal range
Isovalerylcarnitine (C5)	3.64 μmol/L	< 0.90 μmol/L
Ratio of isovalerylcarnitine/butyrylcarnitine (C5/C6)	68.46	< 1.50

The patient was instructed to start a low-protein diet, restrict leucine-rich foods, and supplementation of L-carnitine tablets (500 mg every 8 hours) along with follow-up with a pediatric endocrinologist. The nutritional diagnosis at the beginning was chronic exacerbated malnutrition, but in subsequent controls, the diagnosis was eutrophic. At all times, the patient's psychomotor and neurological development were adequate for her age.

According to her clinical history, the patient received treatment with L-carnitine for approximately two years; it should be noted that the treatment was irregular due to a lack of adherence to it. Subsequently, medical advice was provided to improve adherence to treatment.

After her diagnosis and treatment, the patient has been hospitalized 13 times for decompensated isovaleric academia (hypoglycemia, moderate dehydration, and oral intolerance), associated with infectious conditions, mainly respiratory. Her last hospitalization was in October 2022, for pneumonia and decompensated isovaleric acidemia.

## Discussion

IVA is an inborn error of leucine metabolism [1-3], with a high mortality rate of 33% and, in cases of early onset of symptoms, neurocognitive developmental impairment [3,6].

The clinical presentation is variable and ranges from asymptomatic to severe [2,4]. Three phenotypes have been described: acute neonatal, chronic intermittent, and asymptomatic [2,6,8]. The acute neonatal phenotype is the most severe, which presents within the first few days of birth, with symptoms such as poor feeding, vomiting, acidosis, ketosis, lethargy, and convulsions, which can progress to coma and later death [2,6,8]. The chronic intermittent phenotype, which corresponds to the case of the patient, presents months or years after birth, with recurrent episodes of acidosis and periods of good health between crises; its symptoms are nonspecific and may be accompanied by failure to thrive, developmental delay, and cognitive impairment [2,5,6,8]. This form is associated with catabolic stress events such as infections, surgeries, dehydration, or high protein intake [1,4]. A study by Grünert et al. showed that gastroenteritis is the most common trigger. While high protein intake or surgery did not play a major role as precipitating factors [7,9]. In the asymptomatic phenotype, the patient has no symptoms, but biochemical alteration of isovaleric acid is observed in NBS with less accumulation of isovaleric acid and its derivatives [6]. In our case, the patient presented the chronic intermittent phenotype and most episodes were triggered by gastrointestinal and respiratory infections. Likewise, poor adherence to supplements in recent years also played an important role.

Some laboratory findings during crises are severe metabolic acidosis with elevated anion gap, anemia, neutropenia, thrombocytopenia, or pancytopenia due to bone marrow suppression [2,6,8]. Likewise, hyperammonemia, hyper or hypoglycemia, and hypocalcemia have been reported [6,8]. A characteristic odor of "sweaty feet" has been described during the metabolic crisis due to the accumulation of isovaleric acid in the body, which can be perceived in sweat or cerumen [2,8]. Unlike other acidemias, urine does not have this characteristic odor since unconjugated isovaleric acid is not excreted in the urine in large quantities [2,8]. In the case of our patient, this characteristic odor was not reported.

Clinical diagnosis is based on the detection of isovalerylglycine CoA metabolites in the urine organic acid analyses and elevated levels of isovaleryl (C5) carnitine in blood [1,7] by gas chromatography and mass spectrometry (GC-MS) [6,8,10]. Molecular genetic analysis confirms the diagnosis, as it detects the IVD gene mutation located on chromosome 15q14-15 [2]. However, in this case, no DNA mutations were detected. Other diseases, such as diabetic ketoacidosis characterized by metabolic acidosis, hyperglycemia, ketonuria [8,10], and beta-oxidation defects associated with hyperammonemia should be considered in the differential diagnosis [8].

During the episode of decompensation, supportive treatment, intensive hydration, correction of metabolic acidosis, leucine-free diet, supplementation with L- L-carnitine and L-glycine, and the administration of antibiotics if an underlying infection [1,2,7] is involved are recommended. The most important long-term strategy is the prevention of situations that induce the patient to a catabolic state such as toxic infections [1,4,7,11]. Likewise, a lower protein intake, leucine-free diet, and daily supplementation with L-carnitine and/or L-glycine will improve the conjugation of free isovaleric acid toxicants to non-toxic conjugates [1,4,7,11]. The combination of these two maximizes the excretion of isovaleryl-CoA conjugates, but we still lack studies to determine the clinical benefits of combined or singular supplementation [11]. The recommended dose of L-carnitine is 100 mg per kg per day in three doses and the dose of L-glycine is 100 to 300 mg per kg per day in three doses [6].

Acute catabolic episodes that produce decompensation in these patients are life-threatening, especially during childhood [6]. Therefore, early diagnosis and treatment are necessary to reduce mortality and avoid neurocognitive sequelae [2,6]. Through extended neonatal screening, asymptomatic patients could be diagnosed, thus preventing decompensation and improving cognitive development [2,6].

There is no laboratory marker for monitoring the therapeutic control or status of this disease [8]. However, measurement of plasma-free carnitine concentrations could help determine the need for and monitoring of carnitine supplementation [8]. Nonetheless, age-appropriate weight gain, growth, and development are the best indicators of treatment efficacy, which should be routinely assessed during office follow-up of these patients [3,5].

## Conclusions

IVA is a rare disease, but it should be considered within the differential diagnosis according to the clinical presentation of the patient. In addition, worldwide, including Peru, this disease should be included in NBS due to the importance of early diagnosis of these patients, which is crucial for adequate cognitive development in the long term. On the other hand, adherence to supplements, a low-protein diet, and infection prevention are key points to prevent acute decompensation, so patient and family education is important. However, L-glycine is not available in all countries and its acquisition is often not affordable for the patient. Since there is no laboratory marker during the follow-up of the disease, weight gain, growth, and development play an important role in assessing the efficacy of the treatment.
